# Supplementary material for: Nanobubble-actuated ultrasound neuromodulation for selectively shaping behavior in mice
Source: Nat Commun. 2024 Mar 13;15:2253. doi: 10.1038/s41467-024-46461-y (PMC10937988; doi:10.1038/s41467-024-46461-y)
Supplement: Supplementary file 1 — Supplementary Information [file 41467_2024_46461_MOESM1_ESM.pdf]

Supplementary Information

Nanobubble-actuated ultrasound neuromodulation for selectively shaping behavior in mice

Xuandi Hou[1,3], Jianing Jing[1,3], Yizhou Jiang[1], Xiaohui Huang[1], Quanxiang Xian[1], Ting Lei[1], Jiejun Zhu[1,2], Kin Fung Wong[1], Xinyi Zhao[1], Min Su[1], Danni Li[1], Langzhou Liu[1], Zhihai Qiu[2], and Lei Sun[1*]

[1]Department of Biomedical Engineering, The Hong Kong Polytechnic University, Hung Hom, Hong Kong SAR, P. R. China, 999077

[2]Guangdong Institute of Intelligence Science and Technology, Hengqin, Zhuhai, Guangdong, China, 519031

[3]These authors contributed equally

*Correspondence to Lei Sun (lei.sun@polyu.edu.hk)

Supplementary Figures 1-10, Custom Code, Movie 1-6

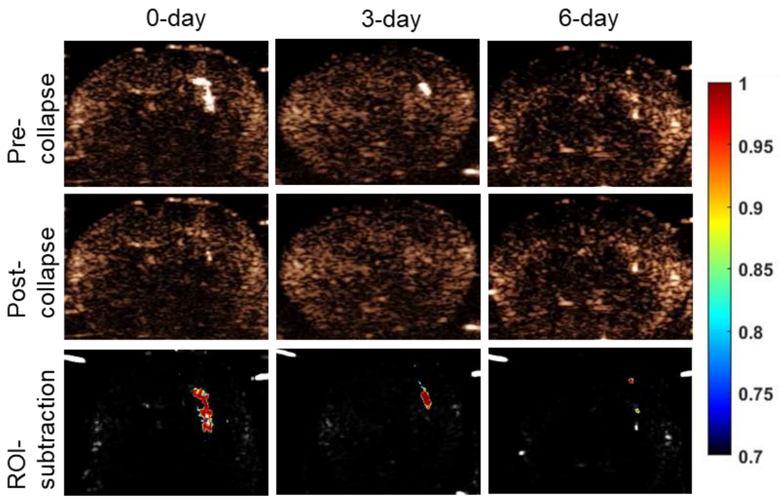

Supplementary Figure 1. Ultrasound images of mice brains. Ultrasound contrast-mode images of GV-injected mice brains. GVs collapsed with destructive insonation. Representative ultrasound images were from n=3 independent experiments.

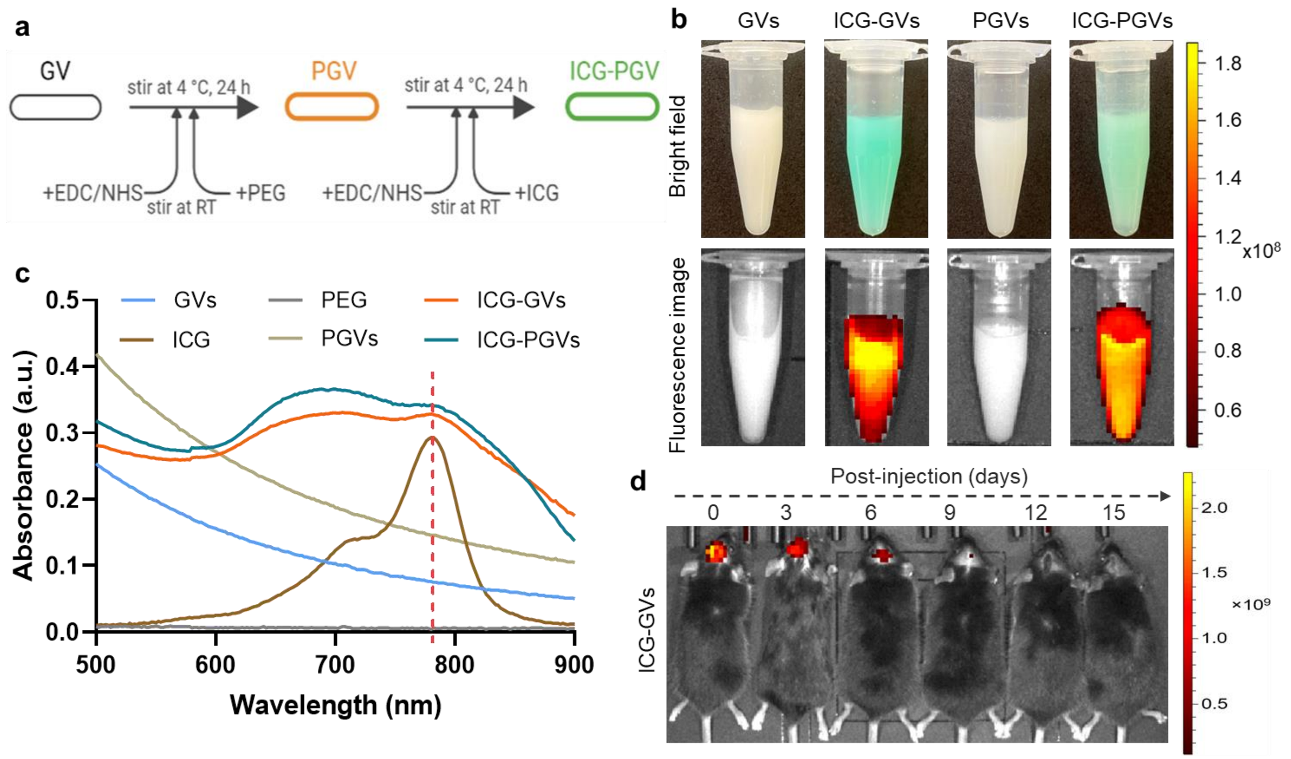

Supplementary Figure 2. GVs surface modification and characterization. a) GVs were coated with PEG to produce PEG-GVs (PGVs, yellow) and modified with ICG to generate ICG-PGVs (green) in EDC/NHS solution. **b**) NIR fluorescence images and bright field images of GVs and modified GVs (ICG-GVs, PGVs, ICG-PGVs). **c**) Absorbance spectrum of PEG, ICG, GVs, and modified GVs. (ICG,

excitation/emission wavelength: 780/805 nm). **d**) NIR fluorescent imaging of mice brains with several injected-time points (0, 3, 6, 9, 12, and 15 days) with brain ICG-GVs injection. Representative images were from n=3 independent experiments in panels (**b**) and (**d**).

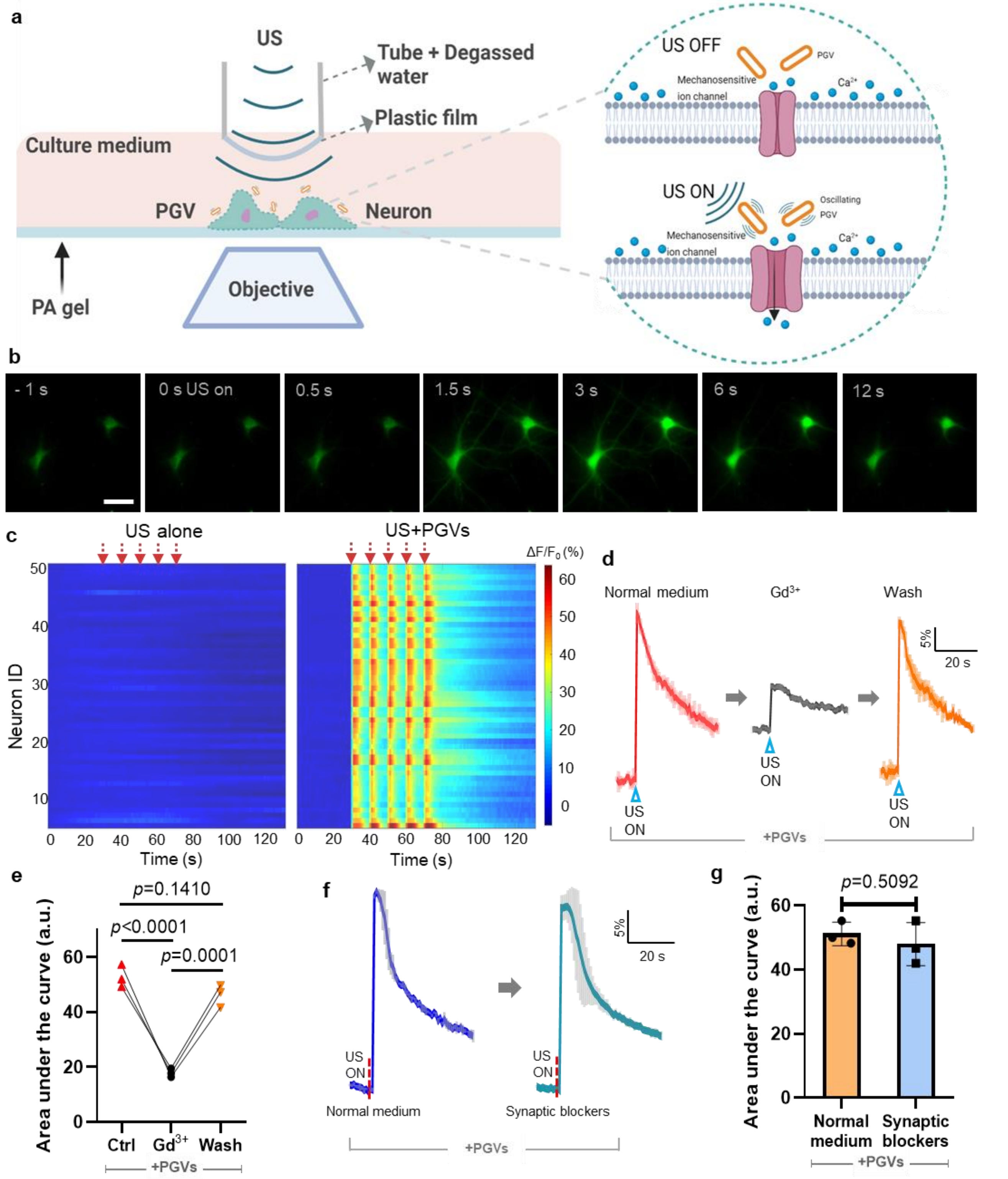

Supplementary Figure 3. Ultrasound mechanically activate neurons via PGVs. a) Illustration of the PGVs+US stimulation setup. Ultrasound waves (1.0 MHz) via plastic film-contained degassed

water are delivered to Fluo 4-stained primary neurons cultured on an acoustically transparent PA-gel, while the neuronal calcium response is recorded by live-cell imaging. Enlarged panel (right) showing ultrasonic activation of mechanosensitive ion channels in the plasma membrane of cells mediated by PGVs. **b**) Representative time-lapse images of Fluo 4 fluorescence before, during, and after one US pulse in the presence of PGVs (0.2 MPa, 300 ms pulse duration, onset at 0 s). Images represent 3 independent experiments. Scale bars, 50 μm. **c**) Heatmap of the normalized Ca^{2+} fluorescence intensity change ($\Delta F/F_0$) of 50 randomly selected primary neurons from 5 independent trials in the control US-alone group (left panel), and the PGVs+US group (right panel). Red arrows indicate US onsets (five US pulses). **d**) Representative Ca^{2+} imaging time courses of neurons treated with PGVs+US, before, during, and after Gd^{3+}. **e**) Average calcium response under each condition (n=3 independent experiments, one-way ANOVA with post-hoc Tukey test). **f**) Representative Ca^{2+} imaging time courses of calcium responses before and after treatment with synaptic blockers NBQX and gabazine. The mean trace is solid and SD is shaded in panels (**d**) and (**f**). **g**) Quantification of the area under the curve before and after adding synaptic blockers (n=3 independent experiments, two-tailed unpaired t-test), a.u., arbitrary units. Data are presented as mean ± SEM.

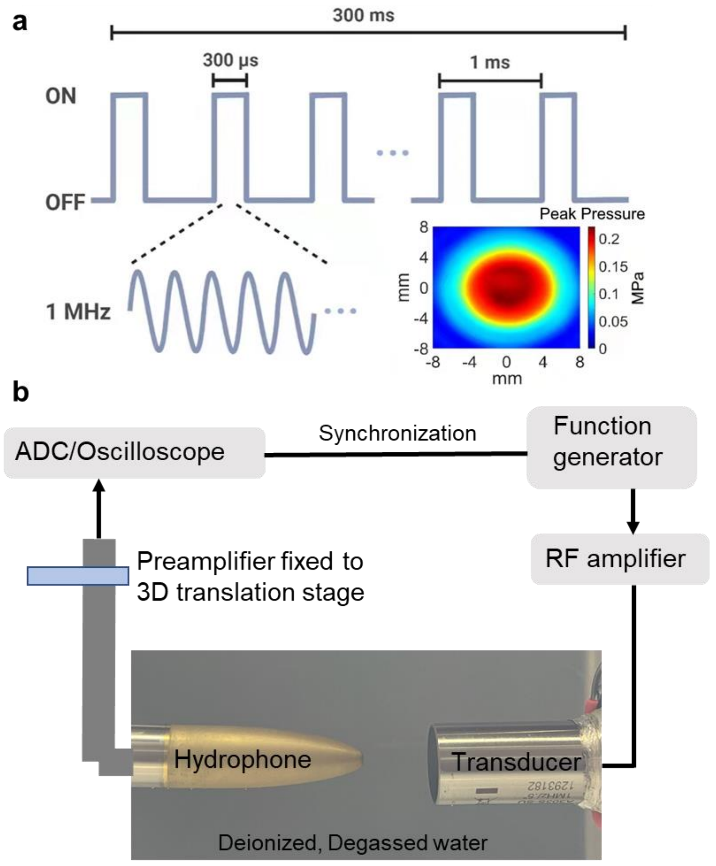

Supplementary Figure 4. Ultrasound acoustic field test. a) Schematic of the acoustic waveform applied to neurons and representative focal pressure waveform measured by a hydrophone. The color map shows the spatial profile of the acoustic pressure. **b**) System for the acoustic field test (1.0 MHz transducer). **c**) A scheme of passive cavitation detection with a hydrophone for calculating the backscattered ultrasonic signals of cavitation under PGVs+US stimulation.

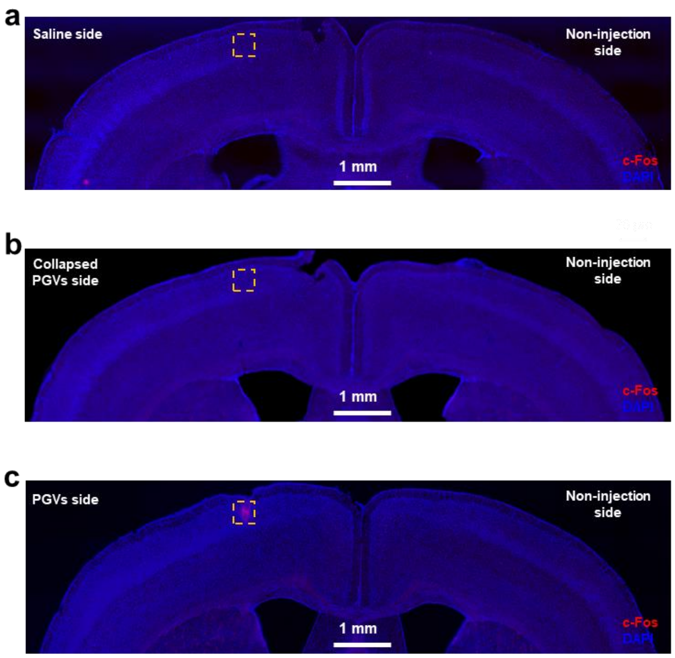

Supplementary Figure 5. c-Fos expression in mouse cortex. Low-magnification image of mice brains expressing c-Fos, showing the pattern of c-Fos expression in mice brains with saline (**a**), collapsed PGVs (**b**), and PGVs (**c**) and treated with US. The cortical injection site is indicated by the yellow dotted squares. Scale bars represent 1 mm. n=9 for the $PGVs^+$ group, n=8 for the Saline and Collapsed PGVs-injected group. Images represent 8 independent experiments.

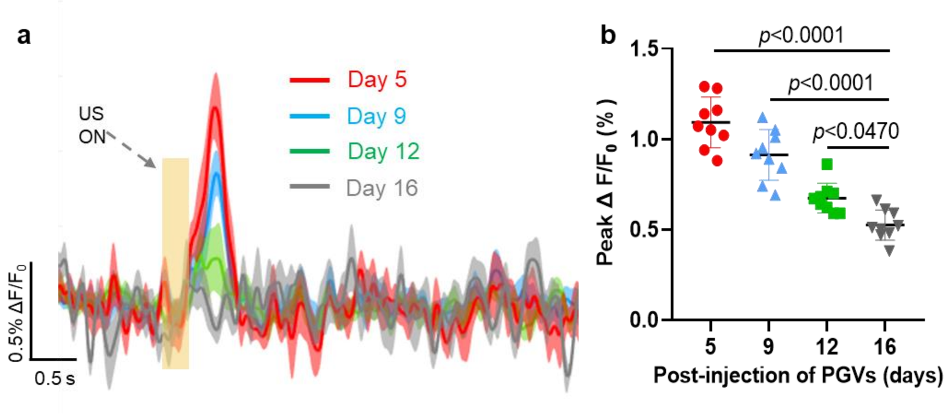

Supplementary Figure 6. Fiber photometry recorded neural Ca^{2+} responses in mice Striatum. a) Mean jRGECO1a fluorescence (Ca^{2+} signals) traces $PGVs^{+}$ mice under US stimulation at days 5, 9, 12, and 16. The mean trace is solid and SD is shaded. **b**) Mean peak of Ca^{2+} responses under 0.09 MPa US irradiation at several injected days (n=9 mice). One-way ANOVA with Tukey's multiple comparisons test. Data are presented as mean ± SD.

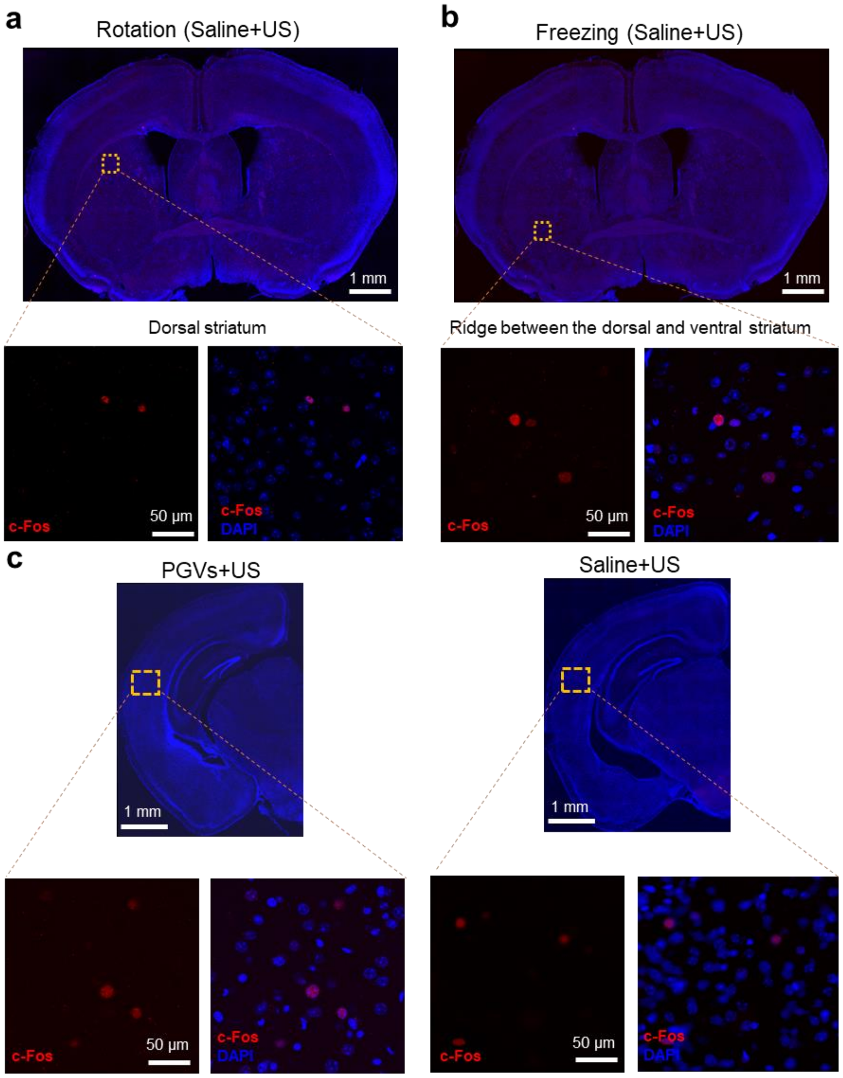

Supplementary Figure 7. c-Fos expression in mice's different brain regions. Representative pictures of c-Fos immunohistochemistry in the Dorsal striatum (**a**) and Ridge between the dorsal and ventral striatum (**b**) of mice treated by Saline+US. **c**) Representative pictures of c-Fos immunohistochemistry in the auditory cortex of PGVs+US-treated mice (left) and Saline+US-treated mice (right). n=8 for the Saline+US group in (**a**), and (**b**), n=8 for the Saline+US group, and n=9 for the PGVs+US group in (**c**). Images represent 8 independent experiments in panels (**a**)-(**c**).

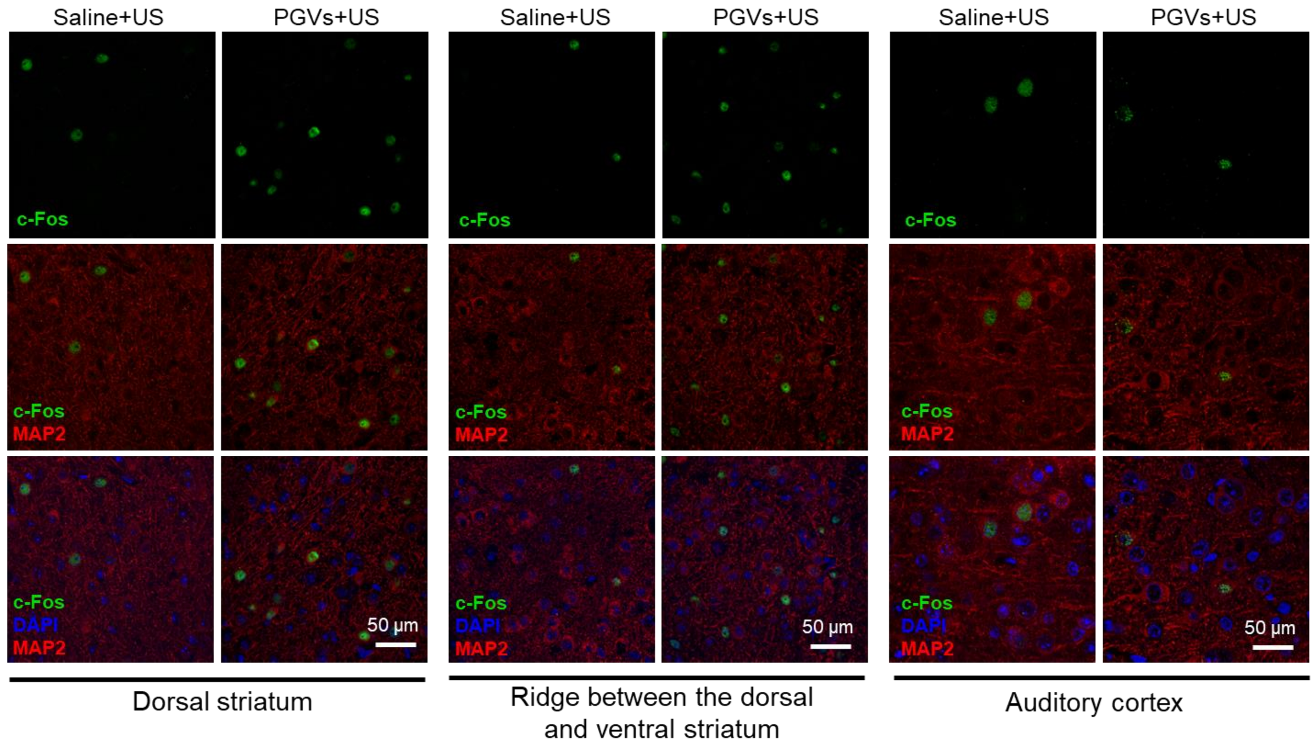

Supplementary Figure 8. Enlarged c-Fos expression in different brain regions. Representative images of different brain regions treated with ultrasound and Saline/PGVs, stained for c-Fos expression. n=8 mice for Saline+US group and n=10 for PGVs+US group in Dorsal striatum, n=8 for Saline+US group and n=9 for PGVs+US group in Ridge between the dorsal and ventral striatum, n=8 for Saline+US group and n=9 for PGVs+US group in Auditory cortex. Images represent 8 independent experiments.

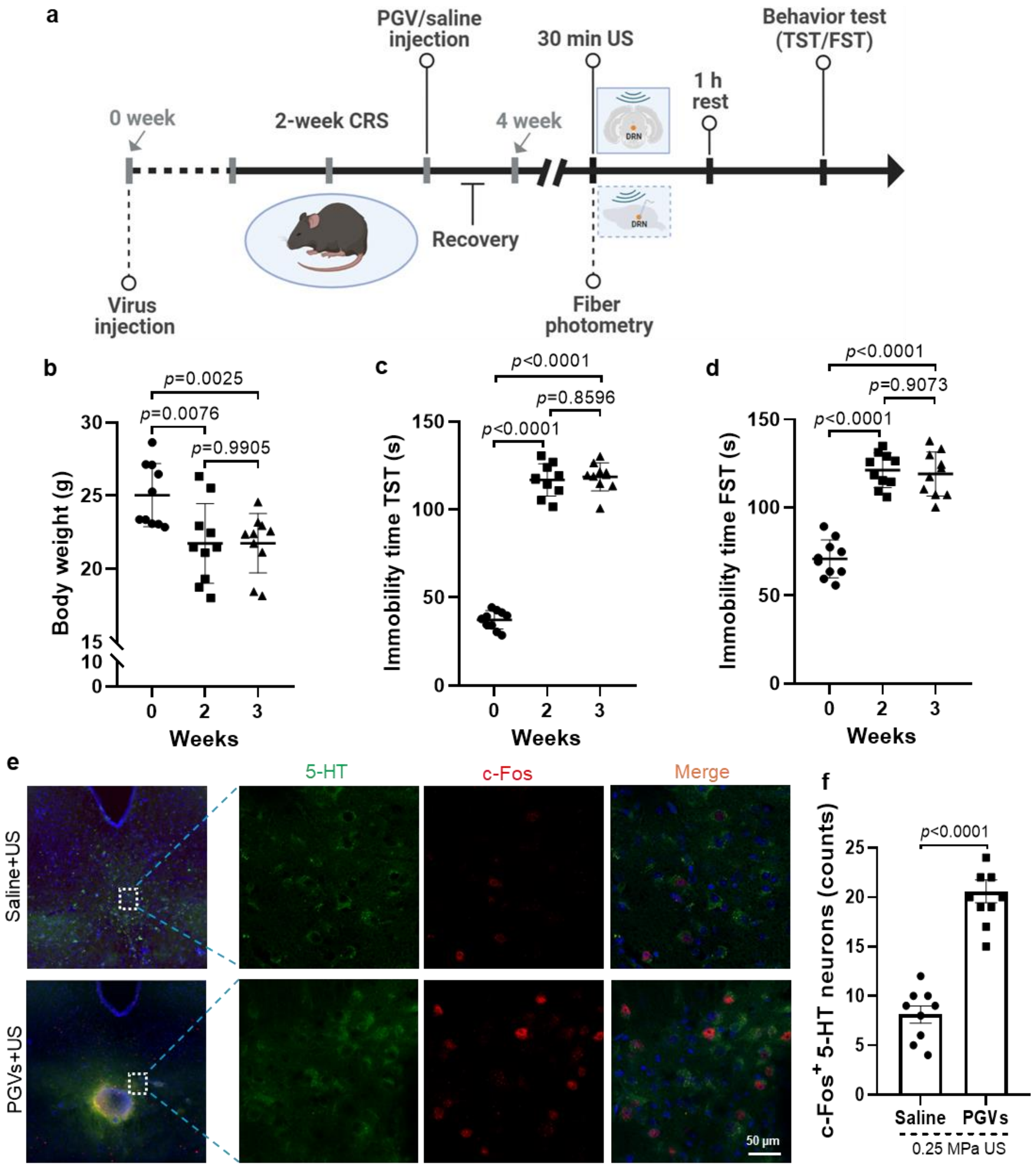

Supplementary Figure 9. Depression-like mice modeling and treatment. a) Timeline of our experimental regimen for the targeted stimulation of the serotonergic dorsal raphe nucleus (DRN). **b**) Body weights of mice before and after CRS depression model establishment. Immobility time before and after model establishment in TST (**c**) and FST (**d**). For panels (**b**), (**c**) & (**d**), one-way ANOVA with post-hoc Tukey test. n=10 mice. **e**) Immunofluorescence of c-Fos^{+} cells (red) in mouse DRN with

the expression of a 5-HT sensor (green). Images represent 9 independent experiments. **f**) c-Fos$^+$ neurons counted around the 5-HT sensor region in the DRN of Saline$^+$ mice (n=9) and PGVs$^+$ mice (n=10) after ultrasound stimulation, unpaired 2-tailed t-test.

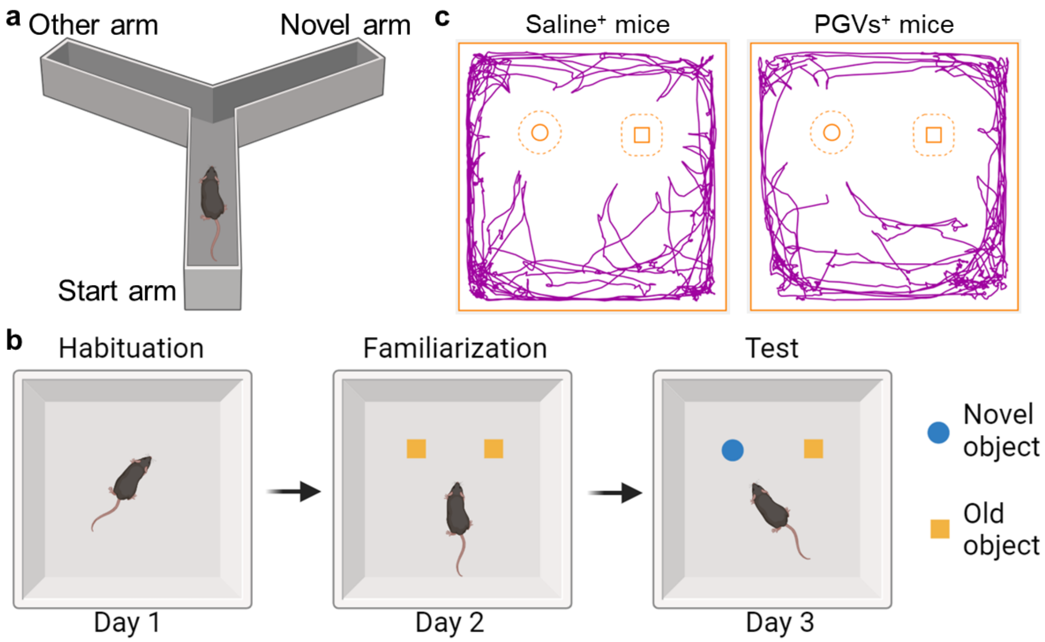

Supplementary Figure 10. PGVs biosafety test. a) Schematic illustration of the Y-maze test. **b**) Schematic showing the novel object recognition (NOR) of Saline$^+$/PGVs$^+$ mice, including the habituation on day 1, familiarization on day 2, and final test on day 3. **c**) Representative trajectories recorded from Saline$^+$ mice (left) and PGVs$^+$ mice (right) during the NOR test.

Custom code for neuronal calcium signals analysis:

```
%% Define the parameter

%b=16;%the column of background

c=1;%the frame rate of camera.

d=?;%number of neurons.

nn=?;%number of files to be processed


a=4*d-1;%column the last signal.
```

```
% %background subtraction;
% for i=1:1:a;
%     num_bs(:,1)=num(:,1);
%     num_bs(:,i)=num(:,i)-num(:,b);
% end


for mm =1:nn %loop to get all data
    %% open file from path;
[fname,fpath] = uigetfile({'*.xlsx';'*.xls'});
if fpath==0, error('no file selected'); end
[num,txt,raw] = xlsread(fullfile(fpath,fname));
%% delatF/F0(%)
num_bsn(:,1,mm)=num(:,1);
for i=3:4:a
    num_bsn(:,i,mm)=(num(:,i)-num(1,i))*100/num(1,i);
end
fprintf([fname 'read\n']);
end
maxi=max(max(max(num_bsn(:,3:4:a,:))));
mini=min(min(min(num_bsn(:,3:4:a,:))));


for mm =1:nn %loop to draw graph
%% plot
```

```
figure;
hold on;
for i=3:4:a
plot((num_bsn(:,1,mm)-num_bsn(1,1,mm))/c,num_bsn(:,i,mm));
end
title([num2str(mm) ':line10']);%change the title
ylim([mini maxi]);
ylabel('{\Delta}F/F0(%)');
xlabel('Time(s)');
%% statistical graph
figure;
imagesc((num_bsn(:,1,mm)-num_bsn(1,1,mm))/c,1:d,num_bsn(:,3:4:a,mm)',[mini maxi]);
colormap(jet);
set(gca, 'YDir', 'normal');
title([num2str(mm) ':map10']);%change the title
ylabel('Neuron ID');
xlabel('Time(s)');
h=colorbar;
set(get(h,'title'),'string','{\Delta}F/F0(%)');
%% adjust threshold and generate new line figure
numnew=[];
x=[];
x=input('threshold?');
```

```
if ~isempty(x)
j=1;
for i=1:1:a
    if any(num_bsn(:,i,mm)>x)
        numnew(:,j)=num_bsn(:,i,mm);
    end
    j=j+1;
end
numnew(:,all(numnew==0,1)) = [];
[m,n]=size(numnew);
figure;
hold on;
for i=1:n
plot(num_bsn(:,1,mm)/c,numnew(:,i));
end
title([num2str(mm) ':line10-above threshold']);%change the title
ylabel('{\Delta}F/F0(%)');
xlabel('Time(s)');
%% calculate mean and sd
numnewm=mean(numnew(:,2:end),2);
sd=std(numnew(:,2:end),0,2);
figure;
errorbar(num_bsn(:,1,mm)/c,numnewm,sd);
```

```
title([num2str(mm) ':line10-above threshold-mean\pmsd']);%change the title

ylabel('{\Delta}F/F0(%)');

xlabel('Time(s)');

%% save data to excel file;

%save extracted data that is above the threshold.

extracteddata=[numnew,numnewm,sd];

xlswrite([num2str(mm) '_deltaF-F0.xlsx'], num_bsn(:,:,mm));% change file name

xlswrite([num2str(mm) '_extracteddata.xlsx'], extracteddata);% change file name

end % end of threshold processing

end %end of loop
```
