# Supplementary material for: Nanobubble-actuated ultrasound neuromodulation for selectively shaping behavior in mice
Source: Nat Commun. 2024 Mar 13;15:2253. doi: 10.1038/s41467-024-46461-y (PMC10937988; doi:10.1038/s41467-024-46461-y)
Supplement: Supplementary file 3 — Description of Additional Supplementary Files [file 41467_2024_46461_MOESM3_ESM.pdf]

Description of Additional Supplementary Files

File Name: Supplementary Movie 1
Description: Primary neurons show rapid and reversible calcium influx in response to each ultrasound pulse (0.20 MPa, 5 pulses) in the presence of PGVs (0.8 nM).

File Name: Supplementary Movie 2
Description: Ultrasound stimulation (0.40 MPa) triggers left forelimb movements of the PGV^+ mouse (left), but not in Collapsed PGV (CPGV)-injected mouse (middle), and $Saline^+$ mouse (right).

File Name: Supplementary Movie 3
Description: Ultrasound stimulation of the dorsal striatum (0.56 MPa) evokes anti-clockwise rotation of the PGV^+ mouse (left), but not the $Saline^+$ mouse (right). Movie speed is 3 × accelerated, PGV/Saline injection location is marked within the movie frames.

File Name: Supplementary Movie 4
Description: Ultrasound stimulation of the deep striatum (0.56 MPa) causes freezing of the PGV^+ mouse (left), but not the $Saline^+$ mouse (right). Movie speed is 3 × accelerated, PGV/Saline injection location is marked within the movie frames.

File Name: Supplementary Movie 5
Description: Ultrasound stimulation of dorsal raphe nucleus (0.25 MPa) improves the struggling time of the tail suspension test of the PGV^+ mouse (left), but not the $Saline^+$ mouse (right). Movie speed is 3 × accelerated.

File Name: Supplementary Movie 6
Description: Ultrasound stimulation of dorsal raphe nucleus (0.25 MPa) decreases the immobility time of the forced swimming test of the PGV^+ mouse (left), but not the $Saline^+$ mouse (right). Movie speed is 3 × accelerated.
