# Supplementary material for: Nanobubble-actuated ultrasound neuromodulation for selectively shaping behavior in mice
Source: Nat Commun. 2024 Mar 13;15:2253. doi: 10.1038/s41467-024-46461-y (PMC10937988; doi:10.1038/s41467-024-46461-y)
Supplement: Supplementary file 10 — Reporting Summary [file 41467_2024_46461_MOESM10_ESM.pdf]

nature portfolio

Corresponding author(s): Lei SUN

Last updated by author(s): Jan 17, 2024

Reporting Summary

Nature Portfolio wishes to improve the reproducibility of the work that we publish. This form provides structure for consistency and transparency in reporting. For further information on Nature Portfolio policies, see our Editorial Policies and the Editorial Policy Checklist.

Statistics

For all statistical analyses, confirm that the following items are present in the figure legend, table legend, main text, or Methods section.

n/a	Confirmed	
☐	☒	The exact sample size (n) for each experimental group/condition, given as a discrete number and unit of measurement
☐	☒	A statement on whether measurements were taken from distinct samples or whether the same sample was measured repeatedly
☐	☒	The statistical test(s) used AND whether they are one- or two-sided<br>*Only common tests should be described solely by name; describe more complex techniques in the Methods section.*
☒	☐	A description of all covariates tested
☐	☒	A description of any assumptions or corrections, such as tests of normality and adjustment for multiple comparisons
☐	☒	A full description of the statistical parameters including central tendency (e.g. means) or other basic estimates (e.g. regression coefficient) AND variation (e.g. standard deviation) or associated estimates of uncertainty (e.g. confidence intervals)
☐	☒	For null hypothesis testing, the test statistic (e.g. F, t, r) with confidence intervals, effect sizes, degrees of freedom and P value noted<br>*Give P values as exact values whenever suitable.*
☒	☐	For Bayesian analysis, information on the choice of priors and Markov chain Monte Carlo settings
☒	☐	For hierarchical and complex designs, identification of the appropriate level for tests and full reporting of outcomes
☒	☐	Estimates of effect sizes (e.g. Cohen's d, Pearson's r), indicating how they were calculated

Our web collection on statistics for biologists contains articles on many of the points above.

Software and code

Policy information about availability of computer code

Data collection	The cellSens software was used to acquire fluorescence images. Cell images and brain slices were collected with fluorescence microscope (Nikon Eclipse Ti2-E Live-cell Fluorescence Imaging System, Leica TCS SPE Confocal Microscope). The FUJIFILM VisualSonics Vevo LAZR system was used to acquire the ultrasound imaging data. The Medusa 1.06.01 was used to acquire electromyography data. The FiberPhotometry_dual_color (Thinkertech) software was used to acquire the in vivo calcium responses data. The Perkin-Elmer IVIS Lumina Series III in vivo animal imaging system was used to obtain PGVs lifetime in vivo data. The camera was used to collect mouse behavior data.
Data analysis	Statistical analysis was performed using Microsoft Excel 2016, GraphPad Prism 8.0.1, MATLAB R2020b, ImageJ 1.52c, TRACKER 6.0.2, ANY-maze Video Tracking System 7.20, Custom code in Supplementary information.

For manuscripts utilizing custom algorithms or software that are central to the research but not yet described in published literature, software must be made available to editors and reviewers. We strongly encourage code deposition in a community repository (e.g. GitHub). See the Nature Portfolio guidelines for submitting code & software for further information.

Data

Policy information about availability of data

All manuscripts must include a data availability statement. This statement should provide the following information, where applicable:

- Accession codes, unique identifiers, or web links for publicly available datasets
- A description of any restrictions on data availability
- For clinical datasets or third party data, please ensure that the statement adheres to our policy

The main data supporting the results in this study are available within the paper and its Supplementary Information. The raw and analysed datasets generated during the study are too large to be publicly shared, yet they are available for research purposes from the corresponding author upon reasonable request.

Research involving human participants, their data, or biological material

Policy information about studies with human participants or human data. See also policy information about sex, gender (identity/presentation), and sexual orientation and race, ethnicity and racism.

Reporting on sex and gender	The study did not involve human research participants.
Reporting on race, ethnicity, or other socially relevant groupings	This study did not include human samples.
Population characteristics	This study did not include human samples.
Recruitment	This study did not include human samples.
Ethics oversight	This study did not include human samples.

Note that full information on the approval of the study protocol must also be provided in the manuscript.

Field-specific reporting

Please select the one below that is the best fit for your research. If you are not sure, read the appropriate sections before making your selection.

☒ Life sciences ☐ Behavioural & social sciences ☐ Ecological, evolutionary & environmental sciences

For a reference copy of the document with all sections, see nature.com/documents/nr-reporting-summary-flat.pdf

Life sciences study design

All studies must disclose on these points even when the disclosure is negative.

Sample size	No statistic method was used to predetermine sample size. For most in vitro experiments, at least 3 independent biological replicates were conducted. The sample size of all mouse experiments should be at least 6 for each group. The specific sample size for each experiment is detailed in the figure legend.
Data exclusions	No data were excluded.
Replication	Replicate experiments on independent animals were performed to ensure the reproducibility of the results. Both in vitro and in vivo experiments were replicated in at least three independent experiments. The experimental findings were reliably reproduced.
Randomization	Mice and cells were randomly allocated into different experimental groups.
Blinding	Blinding was applied to all the experiments.

Reporting for specific materials, systems and methods

We require information from authors about some types of materials, experimental systems and methods used in many studies. Here, indicate whether each material, system or method listed is relevant to your study. If you are not sure if a list item applies to your research, read the appropriate section before selecting a response.

Materials & experimental systems

n/a	Involved in the study
☐	☒ Antibodies
☒	☐ Eukaryotic cell lines
☒	☐ Palaeontology and archaeology
☐	☒ Animals and other organisms
☒	☐ Clinical data
☒	☐ Dual use research of concern
☒	☐ Plants

Methods

n/a	Involved in the study
☒	☐ ChIP-seq
☒	☐ Flow cytometry
☒	☐ MRI-based neuroimaging

Antibodies

Antibodies used	Primary antibodies: Chicken anti-MAP2 (PA1-10005, Invitrogen); Rabbit anti-Caspase-3 (#9661, Cell Signaling Technology); Rabbit anti-Iba-1 (#17198, Cell Signaling Technology); Rabbit anti-GFAP (#12389, Cell Signaling Technology); Rabbit anti-TPH2 (#51124, Cell Signaling Technology); Rabbit anti-c-Fos (#2250, Cell Signaling Technology).<br>Secondary antibodies: Goat anti-chicken, Alexa Fluor 555 (A-21103, Invitrogen); Goat anti-rabbit, Alexa Fluor 488 (A-21428, Invitrogen).
Validation	The antibodies were validated in previous work and in our laboratory for immunohistological staining on mouse brain slices (C57BL/6). Specifically, we relied on the references listed on the manufacture's website:<br>1. https://www.thermofisher.com/antibody/product/MAP2-Antibody-Polyclonal/PA1-10005<br>2. https://www.cellsignal.com/products/primary-antibodies/cleaved-caspase-3-asp175-antibody/9661<br>3. https://www.cellsignal.com/products/primary-antibodies/iba1-aif-1-e4o4w-xp-rabbit-mab/17198<br>4. https://www.cellsignal.com/products/primary-antibodies/gfap-d1f4q-xp-rabbit-mab/12389<br>5. https://www.cellsignal.com/products/primary-antibodies/tph2-d3e5i-xp-rabbit-mab/51124<br>6. https://www.cellsignal.com/products/primary-antibodies/c-fos-9f6-rabbit-mab/2250<br>7. https://www.thermofisher.com/antibody/product/Goat-anti-Chicken-IgY-H-L-Cross-Adsorbed-Secondary-Antibody-Polyclonal/A-21103<br>8. https://www.thermofisher.com/antibody/product/Goat-anti-Rabbit-IgG-H-L-Cross-Adsorbed-Secondary-Antibody-Polyclonal/A-21428

Animals and other research organisms

Policy information about [studies involving animals](); [ARRIVE guidelines]() recommended for reporting animal research, and [Sex and Gender in Research]()

Laboratory animals	C57BL/6 mice (8-week old, male, Jackson Laboratory) were used. The animals were group-housed on a 12 h: 12 h light: dark cycle (temperature: 20–25 °C, humidity: 50–65 %) in the Hong Kong Polytechnic University Centralised Animal Facilities (CAF), and fed with food and water ad libitum as appropriate.
Wild animals	The study did not involve wild animals.
Reporting on sex	Only male mice were used in this study.
Field-collected samples	The study did not involve samples collected from the field.
Ethics oversight	All animal experiments were approved by the Animal Subjects Ethics Sub-Committee (ASESC) of the Hong Kong Polytechnic University and were performed in compliance with the guidelines of the Department of Health - Animals (Control of Experiments) of the Hong Kong S.A.R. government.

Note that full information on the approval of the study protocol must also be provided in the manuscript.

Plants

Seed stocks	*Report on the source of all seed stocks or other plant material used. If applicable, state the seed stock centre and catalogue number. If plant specimens were collected from the field, describe the collection location, date and sampling procedures.*
Novel plant genotypes	*Describe the methods by which all novel plant genotypes were produced. This includes those generated by transgenic approaches, gene editing, chemical/radiation-based mutagenesis and hybridization. For transgenic lines, describe the transformation method, the number of independent lines analyzed and the generation upon which experiments were performed. For gene-edited lines, describe the editor used, the endogenous sequence targeted for editing, the targeting guide RNA sequence (if applicable) and how the editor was applied.*
Authentication	*Describe any authentication procedures for each seed stock used or novel genotype generated. Describe any experiments used to assess the effect of a mutation and, where applicable, how potential secondary effects (e.g. second site T-DNA insertions, mosiacism, off-target gene editing) were examined.*
